# Evidence of antibodies against SARS‐CoV‐2 in wild mustelids from Brittany (France)

**DOI:** 10.1111/tbed.14663

**Published:** 2022-07-27

**Authors:** Bernard Davoust, Patrick Guérin, Nicolas Orain, Camille Fligny, Fabien Flirden, Florence Fenollar, Oleg Mediannikov, Sophie Edouard

**Affiliations:** ^1^ Aix Marseille Univ, IRD, AP‐HM, MEPHI Marseille France; ^2^ IHU Méditerranée Infection Marseille France; ^3^ OpenHealth Company Vannes France; ^4^ Aix Marseille Univ, IRD, AP‐HM, SSA, VITROME Marseille France

**Keywords:** Covid‐19, epidemiosurveillance, France, *Martes martes*, *Meles meles*, mustelids, SARS‐CoV‐2, serology, wildlife

## Abstract

In the French region of Brittany, mainly in the department of the Côtes d'Armor, during the first half of 2021, seropositivity for SARS‐CoV‐2 was detected in five wild mustelids out of 33 animals tested (15.6%). Anti‐SARS‐CoV‐2 IgG was detected against at least four out of five recombinant viral proteins (S1 receptor binding domain, nucleocapsid, S1 subunit, S2 subunit and spike) in three pine martens (*Martes martes*) and in two badgers (*Meles meles*) using the automated western blot technique. An ELISA test also identified seropositive cases, although these did not align with western blot results. Although the 171 qPCRs carried out on samples from the 33 mustelids were all negative, these preliminary results from this observational study nevertheless bear witness to infections of unknown origin. The epidemiological surveillance of Covid‐19 in wildlife must continue, in particular with effective serology tools.

## 1 INTRODUCTION

Human infection with a newly identified coronavirus, SARS‐CoV‐2, was reported in China at the end of 2019 (Huang et al., 2020). This pathogenic coronavirus is responsible for the COVID‐19 pandemic which has caused 483 million cases of infection and 6 million deaths. Despite the health measures taken and the extensive use of vaccines in developed countries, SARS‐CoV‐2 continues to spread, particularly due to the appearance of new genetic variants. The precise origin of this virus has not yet been firmly established, but the fact that the coronavirus closest to SARS‐CoV‐2 (BatCoV RaTG13) has been identified in the intermediate horseshoe bat (*Rhinolophus affinis*) enables us to hypothesize that this coronavirus is a zoonotic pathogen (Zhou et al., 2020). It is possible that an animal coronavirus may have crossed the barrier from bats to humans via a different intermediate host species or even a secondary reservoir (Kumar et al., 2021). Today, SARS‐CoV‐2 is transmitted primarily from person to person, and animals do not seem to be important in the spread of Covid‐19 (Maurin et al., 2021). The infection of companion animals (cats, dogs, ferrets), animals raised for fur (mink) and zoo animals (felines, primates, etc.) by infected people has been well described (Fenollar et al., 2021; Jemeršić et al., 2021; Maurin et al., 2021; Pomorska‐Mól et al., 2021; WOAH, 2022). To date, cases of human infection with SARS‐CoV‐2 transmitted from an animal have proved exceptional but have been described on mink farms (Hammer et al., 2021; Oude Munnink et al., 2021). Recent epidemiological studies have shown that wild animals may also be naturally infected with SARS‐CoV‐2 (Fenollar et al., 2021; Maurin et al., 2021). These observations, although currently very limited, raise the possibility of reservoirs of SARS‐CoV‐2 in wild animals with the potential for virus mutation and transmission from wildlife to humans and are nonetheless of great interest in the context of the prevention of Covid‐19 (Delahay et al., 2021). Infection was found in white‐tailed deer (*Odocoileus virginianus*) first in the United States and then in Canada, and this species is a possible reservoir host for SARS‐CoV‐2 (Hale et al., 2022; Palermo et al., 2022; WOAH, 2022). In addition, in feral American mink (*Neovison vison*) from Utah (USA) and Spain, SARS‐CoV‐2 infection has been detected (Aguiló‐Gisbert et al., 2021; Shriner et al., 2021). It is well known that two well‐studied species of mustelids (ferret and mink) are very receptive and sensitive to the point that the ferret has become a useful model of Covid‐19 for experimental infections (Alluwaimi et al., 2020; Boklund et al., 2021). In a farm in western France, mink were infected and then euthanized at the request of the health authority (Anses, 2021). In this context, an observational study was conducted in the French region of Brittany to detect SARS‐CoV‐2 infection and seroprevalence in wild mustelids.

## 2 MATERIALS AND METHODS

### 2.1 Animals and samples

**FIGURE 1:**
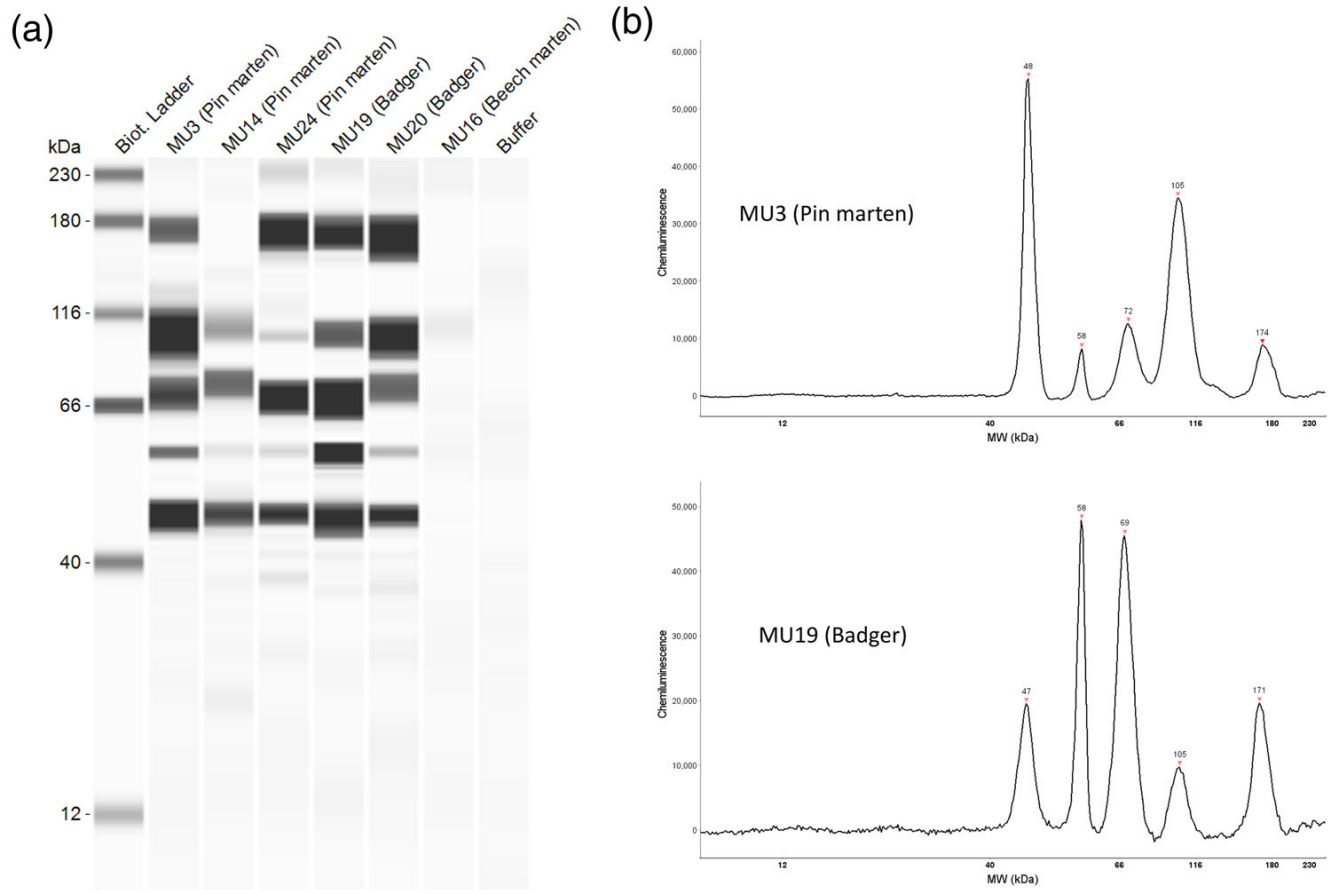
(a) Lane view of automated western immunoblotting including the five positive wild mustelids (MU3, MU14, MU24, MU19, and MU20) and one negative mustelids (MU16). The first lane represents the molecular mass marker in kDa and the last lane the negative control. (b) Chromatogram of chemiluminescence intensity detected by Jess™ Simple Western in the capillaries on positive pine marten (MU3) and on positive European badger (MU19). Bands and peaks were observed for S1 receptor binding domain (RBD) (48 kDa), nucleocapsid (58 kDa), S1 subunit (105 kDa), S2 subunit (71 kDa), and/or spike (170 kDa).

Following an agreement with the hunting federations of two French departments in Brittany, Morbihan and Côtes d´Armor, we were able to take samples from the corpses of 33 mustelids, just after their death. From April to June 2021, we sampled 14 pine martens (*Martes martes*), 10 badgers (*Meles meles*), four American mink (*Neovison vison*), three polecats (*Mustela putorius*) and two beech martens (*Martes foina*). The total sample of 33 mustelids collected comprised 19 females and 14 males. There were 23 animals from the department of the Côtes d´Armor and 10 from the Morbihan (Data S1). The sites where the mustelids were found dead or shot were identified (Figure 1).

In the field, we performed nasal, skin and rectal swabs and blood sampled from the heart (one tube of blood with EDTA and one dry tube with serum separator gel). These samples were transported at +4°C to the laboratory (IHU Méditerranée Infection, Marseille) in less than 48 h. The carcasses of nine mustelids were stored frozen at –20°C in Brittany. Subsequently, the carcasses were transported to the IHU for autopsy and sampling (i.e. lung, spleen, intestine, brain, blood, faeces, nasal swab and lymph nodes).

### 2.2 Serological detection

#### 2.2.1 ELISA assay

For ELISA, we used ID Screen^®^ SARS‐CoV‐2 Double Antigen Multi‐species (Innovative Diagnostics, Grabels, France) following the manufacturer's instructions. The test targets multispecies (i.e. mink, ferrets, cats, dogs, cattle, sheep, goats, horses and all other receptive species) total antibodies (IgG, IgM and IgA) directed against the major nucleocapsid protein of SARS‐CoV‐2. Plates were sensitized with a purified recombinant N antigen. Optical density (OD) was measured at 450 nm using Multiskan GO software (Thermo Scientific, Waltham, MA, USA). The test was validated when the OD of positive control (OD_PC_) was ≥0.35 and a mean ratio of positive (OD_PC_) and negative (OD_NC_) control was higher than 3. The OD of each sample (OD_N_) was used to calculate the sample to positive ratio (S/P) (expressed as a %) where S/P = 100 × (OD_N_ – OD_NC_)/(OD_PC_ – OD_NC_). When the S/P score was lower than 50% by ELISA, samples were considered negative. They were considered as positive when it was higher than 60% and doubtful when 50% < S/P score < 60%.

#### 2.2.2 SARS‐CoV‐2 antigen preparation and automated western immunoblotting (AWB) assay

The Jess™ Simple Western automated nano‐immunoassay system (ProteinSimple, San Jose, CA, USA, a Bio‐Techne Brand), with 25 capillary‐based size separation of proteins, was used to evaluate the absolute serological response to five viral antigens from sera (Edouard et al., 2021). SARS‐CoV‐2 Multi‐Antigen Serology Module^®^ including S1 receptor binding domain (RBD) (48 kDa), nucleocapsid (58 kDa), S1 subunit (105 kDa), S2 subunit (71 kDa) and spike (170 kDa) recombinant proteins as antigens (ProteinSimple) and the 12–230‐kDa Jess separation module (SM‐W004) were used according to the manufacturer's recommendations. We tested up to 21 sera per run in addition to a ladder (molecular weight marker), one human SARS‐CoV‐2 positive serum sample and two negative controls (SARS‐CoV‐2 seronegative human serum and a saturation buffer). Viral protein migration was performed through the separation matrix at 375 volts and immobilized using photoactivated capture chemistry within the ProteinSimple proprietary system. Sera diluted at 1:2 were incubated for 60 min followed by a wash step and underwent 30‐min incubation within an anti‐ferret IgG conjugate (Abcam, Cambridge, UK) diluted to 1/200. Peroxide/luminol‐S (ProteinSimple) was used for the chemiluminescent stage. The Compass Simple Western software (version 6.0.0, ProteinSimple) was used to capture the digital image of the capillary chemiluminescence and analyse the results. A seropositive result with regard to SARS‐CoV‐2 was defined by an AWB Jess™ Covid‐19 test showing reactivity against at least four out of the five recombinant proteins characteristic of SARS‐CoV‐2. For the polecat (MU4), ​​the AWB test was not performed due to insufficient serum.

#### 2.2.3 Biomolecular detection

Viral RNA extraction was performed on an EZ1 Advanced XL device using the EZ1 virus mini kit V2.0 according to the manufacturer's recommendations (Qiagen, Courtaboeuf, France). The qPCR was run on a Lightcycler^®^ 480 thermocycler (Roche diagnostics, Mannheim, Germany) using real‐time fluorescent RT‐PCR kit for 2019‐nCoV (BGI genomics, Hong Kong, China) targeting ORF1ab gene. Positive (pseudo‐virus with target virus gene) and negative (DNAse/RNase free water) controls were added in each qPCR run to validate RNA amplification and absence of RT‐PCR contamination, respectively. Phage RNA internal control was added in each sample to validate RNA extraction (Amrane et al., 2020). This qPCR test was performed on the nasal, rectal and cutaneous swabs and blood sample collected from the 33 mustelids and on biopsies taken from the nine frozen carcasses.

## 3 RESULTS AND DISCUSSION

Our study identified positive ELISA results in 4 out of 33 (12.12%; 95% confidence interval [CI]: 0.99, 23.26) serum samples and positive AWB results in 5 out of 32 (15.6%; 95% CI: 3.04, 28.21) serum samples (Table 1). Five mustelids (four females and one male) were seropositive by AWB. Three martens (MU3, 14, and 24) and two badgers (MU19 and 20) showed high reactivity for RBD, nucleocapsid, S1 subunit, S2 subunit and/or spike (Figure 1). These two badgers were killed at the same place, in Perret (Côte d'Armor), on the same day in May 2021. The two positive martens were from the Côte d'Armor and the third came from the Morbihan (Data S1). In addition, two of the five AWB positive mustelids (pine martens MU3 and 14) were also ELISA positive. Serum from eight mustelids showed reactivity against only one or two proteins out of five viral proteins targeted by AWB. The badger MU10 showed reactivity only against the nucleocapsid protein; the polecat MU32 and the pine marten MU13 showed reactivity only against the RBD. The beech marten MU7, the pine marten MU25 and the badger MU17 showed reactivity against both spike and S2 protein, the American mink MU29 against S1 and S2 subunits and the pine marten MU15 only against RBD and nucleocapsid proteins. Furthermore, there were four ELISA positive animals, only two of which were also positive on AWB (MU11 and MU13).

**TABLE 1:** Results of SARS‐CoV‐2 ELISA, automated western immunoblotting and qPCR of 33 wild mustelids from Brittany (France)

						Western blot							
Animal species	Animal Id.	Gender	Location (Department)	Date of sample	ELISA results	RBD	N	S2	S1	Spike	Conclusion	qPCR performed on field samples: blood, nasal, rectal and skin swabs	qPCR performed on autopsy samples: blood, nasal swab, lung, spleen, intestine, brain, lymph nodes, faeces
Pine marten (*Martes martes*)	MU1	M	Saint‐Avé (56)	04.2021	Neg.	0	0	0	0	0	Neg.	Neg.	NE
	MU2	F	Baden (56)	04.2021	Neg.	0	0	0	0	0	Neg.	Neg.	NE
	MU3	M	La Gacilly (56)	05.2021	Pos.	Pos.	Pos.	Pos.	Pos.	Pos.	Pos.	Neg.	Neg.
	MU5	F	Kergrist (56)	05.2021	Neg.	0	0	0	0	0	Neg.	Neg.	NE
	MU8	M	Noyal‐Pontivy (56)	06.2021	Neg.	0	0	0	0	0	Neg.	Neg.	NE
	MU9	M	Ploeren (56)	06.2021	Neg.	0	0	0	0	0	Neg.	Neg.	NE
	MU13	F	Ploeuc‐L'Hermitage (22)	04.2021	Neg.	Pos.	0	0	0	0	Doubtful	Neg.	NE
	MU14	F	St Brandan (22)	04.2021	Pos.	Pos.	Pos.	Pos.	Pos.	0	Pos.	Neg.	Neg.
	MU15	F	Henon (22)	04.2021	Neg.	Pos.	Pos.	0	0	0	Doubtful	Neg.	NE
	MU21	F	Languédias (22)	06.2021	Neg.	0	0	0	0	0	Neg.	Neg.	NE
	MU24	F	Pedernec (22)	06.2021	Neg.	Pos.	Pos.	Pos.	Pos.	Pos.	Pos.	Neg.	Neg.
	MU25	M	Ploeuc L'Hermitage (22)	06.2021	Neg.	0	0	Pos.	0	Pos.	Doubtful	Neg.	Neg.
	MU26	M	Languédias (22)	06.2021	Neg.	0	0	0	0	0	Neg.	Neg.	NE
	MU33^a^	F	Quemper‐Guézennec (22)	06.2021	Pos.	0	0	0	0	0	Neg.	Neg.	Neg.
European badger (*Meles meles*)	MU10	F	Cleguerec (56)	06.2021	Neg.	0	Pos.	0	0	0	Doubtful	Neg.	NE
	MU11^a^	F	Minihy‐Treguier (22)	04.2021	Pos.	0	0	0	0	0	Neg.	Neg.	NE
	MU12	F	Minihy‐Treguier (22)	04.2021	Neg.	0	0	0	0	0	Neg.	Neg.	NE
	MU17	F	Glomel (22)	03.2021	Neg.	0	0	Pos.	0	Pos.	Doubtful	Neg.	Neg.
	MU18	M	Yvias (22)	05.2021	Neg.	0	0	0	0	0	Neg.	Neg.	NE
	MU19	F	Perret (22)	05.2021	Neg.	Pos.	Pos.	Pos.	Pos.	Pos.	Pos.	Neg.	NE
	MU20	F	Perret (22)	05.2021	Neg.	Pos.	Pos.	Pos.	Pos.	Pos.	Pos.	Neg.	NE
	MU22	M	Ploezal (22)	06.2021	Neg.	0	0	0	0	0	Neg.	Neg.	NE
	MU27	M	Minihy‐Treguier (22)	04.2021	Neg.	0	0	0	0	0	Neg.	Neg.	NE
	MU28	F	Minihy‐Treguier (22)	04.2021	Neg.	0	0	0	0	0	Neg.	Neg.	NE
American mink (*Neovison vison*)	MU23	M	Perros‐Guirec (22)	06.2021	Neg.	0	0	0	0	0	Neg.	Neg.	NE
	MU29	M	Pommerit‐Jaudy (22)	04.2021	Neg.	0	0	Pos.	Pos.	0	Doubtful	Neg.	Neg.
	MU30	M	Pommerit‐Jaudy (22)	04.2021	Neg.	0	0	0	0	0	Neg.	Neg.	NE
	MU31^a^	F	Plédéliac (22)	04.2021	Neg.	0	0	0	0	0	Neg.	Neg.	Neg.
European polecat (*Mustela putorius*)	MU4	M	Cournon (56)	05.2021	Neg.	NE						Neg.	NE
	MU32^a^	F	Plaintel (22)	04.2021	Neg.	Pos.	0	0	0	0	Doubtful	Neg.	Neg.
	MU6	M	Saint‐Gonnery (56)	05.2021	Neg.	0	0	0	0	0	Neg.	Neg.	NE
Beech marten (*Martes foina*)	MU7	F	Arradon (56)	05.2021	Neg.	0	0	Pos.	0	Pos.	Doubtful	Neg.	NE
	MU16	F	Ploumagoar (22)	04.2021	Neg.	0	0	0	0	0	Neg.	Neg.	NE

Abbreviations: NE, not evaluated; Neg., negative; Pos., positive.

^a^ Hemolysed sera could affect the migration of the sample in capillaries of the AWB.

All the swabs (nasal, rectal and cutaneous) and the blood samples taken in the field from the 33 mustelids were negative to the specific SARS‐CoV‐2 RT‐PCR test. Likewise, all the RT‐PCRs carried out on the samples taken from nine carcasses kept frozen were negative.

Initially, serological screening was carried out using the ELISA test. Due to the positivity of several sera, additional investigations were implemented with the AWB to confirm SARS‐CoV‐2 seropositivity. AWB detects IgG against five different viral antigens (including spike subunit and nucleocapsid proteins) and therefore is of higher specificity than the ELISA which only detects total immunoglobulin against the nucleocapsid protein. AWB is a reliable technique to confirm the presence of SARS‐CoV‐2 antibodies and has been successfully employed on human and dog sera (Edouard et al., 2021; Laidoudi et al., 2021). As a secondary antibody, we used an anti‐ferret IgG that may cross‐react with IgG from other closely related species as indicated by the manufacturer. In fact, we found three positive martens and two badgers suggesting that anti‐ferret IgG can also detect marten and badger IgG antibodies efficiently but it is possible that seroprevalence was underestimated in our study. The strong serological reactivity against four or five different antigens of the virus using AWB confirms specificity of antibodies, indicating a humoral immune response linked to contact with the agent of the Covid‐19 pandemic for five mustelids. We did not research the presence of recent infection (by anti‐SARS‐CoV‐2 IgM and IgA detection) and were unable to confirm our result using a neutralization assay because of the low quantity of sera available.

For eight mustelids, AWB results were inconclusive and therefore were not considered positive for SARS‐CoV‐2. The AWB profiles showed reactivity against only one or two proteins suggesting cross‐reactivity with a different coronavirus to SARS‐CoV‐2 or an incomplete serological response, that is to say that some individuals produce antibodies only against the spike (or certain subunits) or the nucleocapsid but not against all five viral antigens (Li & Li, 2021; Lv et al., 2020). Unfortunately, cross‐reactivity with another coronavirus could not be evaluated by AWB because pre‐pandemic mustelid sera were not available. However, cross‐reactivity was detected using AWB and recombinant antigen on pre‐pandemic human sera, but a low value of chemiluminescence was observed and reactivity could be seen for only one to three antigens out of the five (data not shown). Therefore, we chose stringent criteria to define western blot positivity and selected sera as positive if they were reactive against at least four antigens. The ELISA kit uses a truncated nucleocapsid protein in order to limit cross‐reactions with other coronaviruses (Spada et al., 2021). The diagnostic specificity of this test based on double antigens is >99% in dogs (Laidoudi et al., 2021). To date, little data are available on the presence of coronaviruses in wild mustelids. Apart from SARS‐CoV‐2 detected since 2020, there have only been two known coronaviruses, belonging to the *Minacovirus* subgenus of the *Alphacoronavirus* genus (Stout et al., 2021) described in mink farms and in pet ferrets. No coronavirus (other than SARS‐CoV‐2) has been demonstrated in pine martens, badgers, polecats and beech marten.

Dissonant results were found between ELISA and AWB in five mustelids. Discrepancies in the results of the different serological approaches are likely related to the choice of antigens (i.e., specific target, whole antigens), the nature of detected antibodies (total immunoglobulin, IgG, IgM, IgA) and the technique used (ELISA, CLIA, lateral flow immunoassay, indirect immunofluorescence) which will determines sensitivity/specificity of the test (Van Elslande et al., 2020). The sera from badgers MU11 and MU33, which were positive only by ELISA, were too hemolysed to be able to draw reliable conclusions of AWB because capillaries could be blocked by red blood cells. However, three mustelids presented negative ELISA results despite being positive by AWB (MU19, 20, and 24). These results are consistent with the AWB having greater sensitivity than the ELISA test (Cortes et al, 2006; Vola et al., 2019). All samples taken were negative using the highly sensitive RT‐PCR assay. However, internal controls were positive for all the specimens tested attesting to the good quality of the RNA extraction.

It was not our intention to conduct representative sampling from wild mustelids. Instead, we conducted an opportunistic study of a deliberately small number of wild mustelids, which benefited from the collection of carcasses by hunters acting according to the standards in force (French environmental code) in only two departments of Brittany and for a limited time. Our results are therefore not intended to be representative of the epidemiological situation in Brittany and even less throughout France. Instead, they are indicative that further field epidemiological investigations should be carried out. In order to place our observations in the wider epidemiological context of human infection with SARS‐CoV‐2 in the first half of 2021, it would be useful to consult the official statistics for Covid‐19 cases (ARS Bretagne, 2021; Santé publique France, 2022).

To our knowledge, this is the first evidence of SARS‐CoV‐2 infection in European badgers and pine martens, both of which are common in France. The five species of mustelids involved in our study are all solitary animals except badgers. Intra‐species virus transmission is possible, but human‐mediated transmission (spillover from humans) should also be suspected in each case. Initial transmission of SARS‐CoV‐2 to wild mustelids may have occurred through indirect contact with an infected human through environmental contamination (e.g. wastewater, household waste etc.). All the mustelids studied lived in agricultural areas with human settlements. It is possible that direct transmission amongst mustelids may have occurred, although we have no direct evidence for this. Nevertheless, it is interesting that the two infected badgers (MU19 and 20) were from the same location and that this species is known to be more sociable than other mustelids (Wang, 2011).

Viral circulation amongst mink is rapid and they are highly susceptible to the virus (Shuai et al., 2020). Like humans, they express the angiotensin‐converting enzyme 2 (ACE2) receptor on the cells of the respiratory tract, which facilitates viral penetration (via the spike protein) and infection, depending on their abundance and distribution (upper vs. lower respiratory tract) (Lean et al., 2021). SARS‐CoV‐2 infection in ferrets (*Mustela putorius furo*) shows that they remain carriers of the virus for 14 days, while the specific antibodies persist for several months (Monchatre‐Leroy et al., 2021). On affected mink farms, infected animals often lacked clinical signs, although coughing and fever were observed in some and excess mortality was reported (Boklund et al., 2021; Pomorska‐Mól et al., 2021).

The seropositive results that we have highlighted indicate that wild mustelids can be infected with SARS‐CoV‐2, which raises the question of their potential to be reservoir hosts. SARS‐CoV‐2 virus mutation has been demonstrated in experimentally infected ferrets (Everett et al., 2021), and has been well documented in a mink farm in Denmark (Hammer et al., 2021; Oude Munnink et al., 2021). Twelve people in contact with mink carrying SARS‐CoV‐2 were infected with an entirely new emerging variant (cluster 5) (Lassaunière et al., 2021). This FVI‐spike variant virus included a combination of four mutations (69‐70‐deltaHV, 453F, 692V and 1229I) (Bayarri‐Olmos et al., 2021; Lassaunière et al., 2021). This outbreak was quickly brought under control but remains a model for understanding the health risk linked to farmed mink as reservoirs of coronaviruses transmissible to humans. However, the distribution and abundance of wild mustelids is very different and the health risks for humans from their infection are unknown. To protect public health, reinforced epidemiological surveillance and biosecurity measures have been taken, at the request of the health authorities, in the three mink farms remaining in France (Anses, 2021). However, our study shows that the virus has infected wild mustelids of two species (pine martens and badgers). It is therefore important to extend our one‐off investigation to an active epidemiological surveillance of mustelids (injured or killed) from carcasses collected by the departmental hunting federations.

The infection of wild animals with SARS‐CoV‐2 has been increasingly studied in North America since the discovery of infected white‐tailed deer (Hale et al., 2022; Palermo et al., 2022; Palmer et al., 2021). Anti‐SARS‐Cov‐2 antibodies were detected in 152 samples (40%) from 2021 using a surrogate virus neutralization test (Chandler et al., 2021). With the identification of cases in wild animals, the World Organisation for Animal Health has proposed recommendations for people working on wild animals (WOAH, 2020).

## 4 CONCLUSION

This study has demonstrated for the first time SARS‐CoV‐2 infection in wild mammals in France. The results suggest the need for further investigations of SARS‐CoV‐2 infection in wildlife in other regions and countries using effective serological methods. From the origin of the virus in China at the end of 2019, until today with the circulation of SARS‐CoV‐2 in the wildlife of other continents, animals have been observed as potential players and their role should not be neglected in the future of the pandemic of Covid‐19. Taking a *One Health* approach will require intense cooperation amongst public health, veterinary and wildlife ecology professionals.

## CONFLICT OF INTEREST

The authors declare no conflict of interest.

## ETHICS STATEMENT

In seven cases, the animals died as a result of road collision, 11 mustelids were shot dead in accordance with current hunting regulations and 15 others were trapped and euthanized in accordance with article R 427‐6 of the French Environment Code. For ethical reasons of biodiversity protection, strict limits were imposed on the number of animals studied.

## Supporting information

SUPPLEMENTARY DATA 1 Location of the five SARS‐CoV‐2 seropositive mustelids.

## Data Availability

The data that support the findings of this study are available from the corresponding author upon reasonable request.
