# Supplementary material for: Evidence of antibodies against SARS‐CoV‐2 in wild mustelids from Brittany (France)
Source: Transbound Emerg Dis. 2022 Jul 27:10.1111/tbed.14663. Online ahead of print. doi: 10.1111/tbed.14663 (PMC9350122; doi:10.1111/tbed.14663)
Supplement: Supplementary file 1 — SUPPLEMENTARY DATA 1 Location of the five SARS‐CoV‐2 seropositive mustelids. [file TBED-9999-0-s001.pdf]

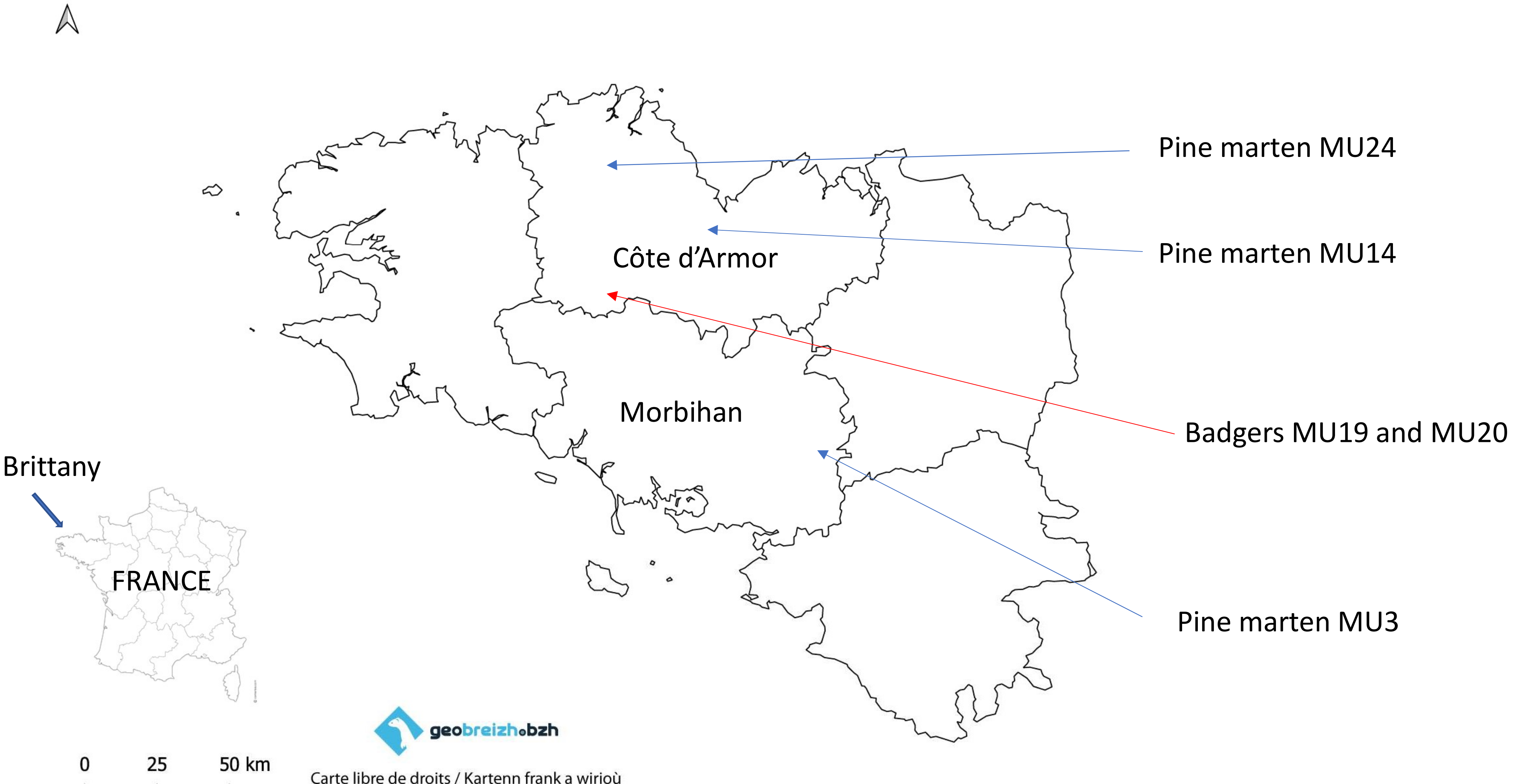

S1. Location of the five SARS-CoV-2 seropositive mustelids
